# Rolling Circle Amplification (RCA)-Mediated Genome-Wide ihpRNAi Mutant Library Construction in *Brassica napus*

**DOI:** 10.3390/ijms21197243

**Published:** 2020-09-30

**Authors:** Shengbo Zhao, Junling Luo, Xinhua Zeng, Keqi Li, Rong Yuan, Li Zhu, Xiaofei Li, Gang Wu, Xiaohong Yan

**Affiliations:** Oil Crops Research Institute of the Chinese Academy of Agricultural Sciences, Key Laboratory of Biology and Genetic Improvement of Oil Crops, Ministry of Agriculture, Wuhan 430062, China; shengbo_ab@163.com (S.Z.); luojunling@caas.cn (J.L.); zengxinhua@caas.cn (X.Z.); likeqi901218@163.com (K.L.); yuanrong4966@163.com (R.Y.); zhuli49751@163.com (L.Z.); lixiaofei@caas.cn (X.L.)

**Keywords:** *Brassica napus*, RNAi, ihpRNA, genome-wide library, mutant, functional genomics

## Abstract

With the successful completion of genomic sequencing for *Brassica napus*, identification of novel genes, determination of functions performed by genes, and exploring the molecular mechanisms underlying important agronomic traits were challenged. Mutagenesis-based functional genomics techniques including chemical, physical, and insertional mutagenesis have been used successfully in the functional characterization of genes. However, these techniques had their disadvantages and inherent limitations for allopolyploid *Brassica napus*, which contained a large number of homologous and redundant genes. Long intron-spliced hairpin RNA (ihpRNA) constructs which contained inverted repeats of the target gene separated by an intron, had been shown to be very effective in triggering RNAi in plants. In the present study, the genome-wide long ihpRNA library of *B. napus* was constructed with the rolling circle amplification (RCA)-mediated technology. Using the phytoene desaturase (*PDS*) gene as a target control, it was shown that the RCA-mediated long ihpRNA construct was significantly effective in triggering gene silence in *B. napus*. Subsequently, the resultant long ihpRNA library was transformed into *B. napus* to produce corresponding RNAi mutants. Among the obtained transgenic ihpRNA population of *B. napus*, five ihpRNA lines with observable mutant phenotypes were acquired including alterations in the floral model and the stamen development. The target genes could be quickly identified using specific primers. These results showed that the RCA-mediated ihpRNA construction method was effective for the genome-wide long ihpRNA library of *B. napus,* therefore providing a platform for study of functional genomics in allopolyploid *B. napus*.

## 1. Introduction

The complete sequencing of the genome of *Brassica napus* [1] has facilitated in the identification of novel genes and their corresponding functions, as well as in the exploration of the molecular mechanisms underlying important agronomic traits. Reverse genetics is an effective approach that has been utilized in functional genomic investigations in plants. Mutagenesis-based functional genomics techniques, including chemical (e.g., sodium azide or ethyl methanesulphonate), physical (e.g., c-radiation), and insertional mutagenesis (e.g., transposons or T-DNAs), have been successfully used in the functional characterization of genes [2,3,4,5,6,7,8]. However, these techniques have disadvantages and inherent limitations. For chemical or physical treatment-based mutagenesis studies, mutations are mapped or sequenced to identify mutation sites, which may be tedious and laborious in the absence of corresponding tags [9]. For insertion mutagenesis using T-DNAs or transposons, although known sequences can be utilized as DNA tags to track down flanking sequences of insertional events, it is generally difficult to generate mutants that harbor all mutations of multigene families [10,11]. To obtain mutants covering genome-wide genes, a large and sufficient mutant population should be produced. Physical, chemical, or insertional mutations are random and usually occur at noncoding and intergenic regions, and these imperfections make it difficult to acquire a sufficient number of mutants. In addition, phenotypes caused by recessive mutations are only manifested in M_2_ populations, thereby further hindering large-scale mutant analysis, and extensive labor and time are necessary to obtain a good coverage of whole genomes [9]. Most importantly, it is highly difficult to obtain mutants of essential genes because functional defects in these genes are often lethal.

*B. napus* (genome A_n_A_n_C_n_C_n_) is an allopolyploid that is generated by the hybridization of *Brassica rapa* (genome A_r_A_r_) and *Brassica oleracea* (genome C_o_C_o_), followed by chromosome doubling [12,13]. Furthermore, the genomes of *B. oleracea* and *B. rapa* share high similarity in nucleotide sequences [14]. Together with more ancient polyploidizations, the genome of *B. napus* has thus accrued a large number of homologous and redundant genes [15]. The *B. napus* genome assembly also contains numerous noncoding sequences, in which 34.8% are transposable elements. RNA sequencing has shown that the genome of *B. napus* possesses about 101,040 gene models, including 91,167 genes that have been confirmed through matches using *B. oleracea* and/or *B. rapa* predicted proteomes [15]. These findings also suggest that the application of the above-mentioned mutagenesis approaches to *B. napus* at the genome-level scale is highly tedious and laborious.

RNA interference (RNAi), also known as post-transcriptional gene silencing (PTGS), which is based on the ability to generate double-stranded RNA (dsRNA), causes targeted knockdown of endogenous genes. RNAi-mediated basic mechanisms of target gene silencing are conserved across kingdoms. dsRNAs are initially processed into 20–24 nt small interfering RNA (siRNA) by the RNase III-like enzyme Dicer [16,17]. The resulting siRNAs are then integrated into RNA-induced silencing complexes (RISCs), which subsequently results in the degradation of target mRNAs by interacting with homologous mRNAs [18]. Compared to insertional/physical/chemical mutagenesis approaches, the RNAi strategy offers the ability to specifically target selected genes, members of gene families and redundant genes, members of a given pathway, and essential genes that are induced by promoters [19,20] and to produce graded levels of knockdowns and knockouts [21]. By these approaches, RNAi offers the flexibility needed in determining gene functions.

RNAi has been successfully utilized as an effective approach in improving disease resistance and food quality of crops [21,22,23,24]. Long intron-spliced hairpin RNA (ihpRNA) constructs that contain homologous antisense and sense inverted repeats of target genes spaced by an intron have been shown to be highly effective in inducing RNAi in plants, with a silencing frequency of >70% [21,24,25]. The rolling circle amplification (RCA)-mediated ihpRNA construction technology facilitates in the efficient and inexpensive construction of genome-wide ihpRNA libraries [26,27]. In a typical RCA, a circular template is isothermally amplified by Phi29 DNA polymerase at 30 °C, generating strands averaging 70 kb in length, which contain a large number of tandem repeats of the starting molecule. These strands in turn serve as templates for new syntheses, thus amplification enters an exponential phase, in which the rate of initiation of new strands is proportional to the quantity of the starting DNA [28,29]. Therefore, RNAi transgenic populations may be generated using RCA-mediated long ihpRNAi library construction that targets all transcripts in plants.

In the present study, the long ihpRNA library of *B. napus* was constructed using the RCA approach. Using the phytoene desaturase (*PDS*) gene as a control target, we determined that RCA-mediated long ihpRNA constructs effectively trigger gene silencing in *B. napus*. Subsequently, the resulting long ihpRNA library was transformed into *B. napus* to produce a RNAi mutant library at a genome-wide scale. Among the obtained transgenic ihpRNA population of *B. napus*, the target genes could be quickly identified using specific primers, and five ihpRNA lines with observable mutant phenotypes were acquired including alterations in the floral model and the stamen development. These results showed that the RCA-mediated ihpRNA construction method was effective for the genome-wide long ihpRNA library of *B. napus,* therefore providing a platform for functional studies of genes on a genome-wide scale in *B. napus*.

## 2. Results

### 2.1. RCA-Mediated ihpRNA Library Construction Procedure

**Figure 1:**
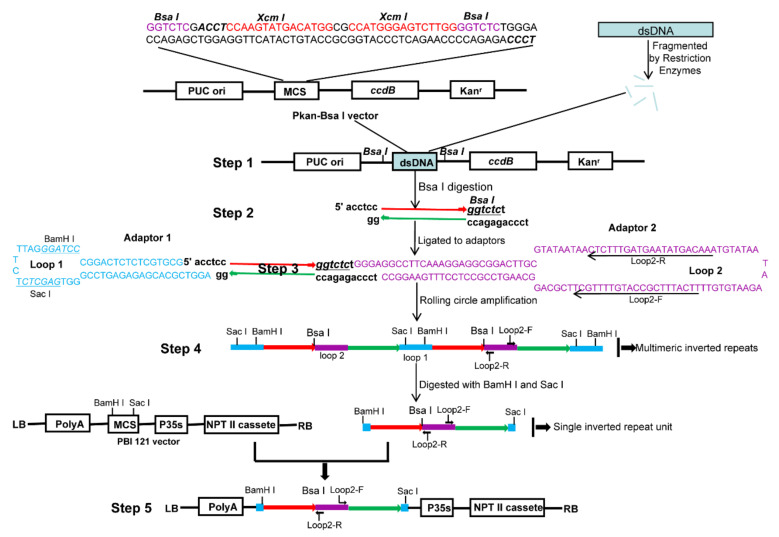
The procedure for RCA-mediated ihpRNA library construction. (Step 1) The Pkan-*Bsa*I vector contains two *Bsa*I sites that were used in generating two different sticky termini in the DNA insertional fragments, two *Xcm*I sites for generating the T-ended vector, and the *ccdB* gene for avoiding the empty vector during the intermediate cDNA library construction process. dsDNAs were fragmented with *Rsa*I, *Bsa*I, *BamH*I, *Sac*I, *Bgl*II, and *Hind*III restriction enzymes, blunted with T4 DNA polymerase, ‘A’ ends were added and ligated to pKan-*Bsa*I. (Step 2) DNA insertion fragments were excised using *Bsa*I, generating *Bsa*I-digested fragments with two asymmetrical sticky termini. (Step 3) The fragments were connected to adaptors 1 and 2 through dissymmetrical sticky ends. Two adaptors carried correspondingly sticky ends complementary to *Bsa*I-digested fragments. (Step 4) RCA was performed to yield a tandem array of double-stranded inverted repeat DNA units by employing the loop2-specific primer pair loop2-F and loop2-R and Phi29 DNA polymerase. (Step 5) RCA products were digested with *Bam*HI and *Sac*I, and the digested fragments were ligated to the eukaryotic expression vector pBI121, generating the final ihpRNA library expression construction. The light blue rectangle is fragmented dsDNA. Blue bases and lines are the adaptor1 sequence. Purple bases and lines are the adaptor2 sequence. Red line with the arrow is the sense strand of dsDNA. Green line with the arrow is the anti-sense strand of dsDNA.

The procedure in generating high-throughput ihpRNA is outlined in Figure 1. The improved intermediate cloning vector Pkan-*Bsa*I was highly efficient for cDNA cloning, containing the ccdB lethal gene and facilitating in the insertion of double strand cDNAs (dscDNAs) (Step 1; Figure 1). DNA insertions were digested, leading to asymmetrical 4-nt 5’ overhangs ‘ACCT’ and ‘TCCC’, respectively, at the two ends, which avoided self-ligation of DNA fragments and tandem ligation between fragments (Step 2; Figure 1). Two different synthetic adaptors (adaptor 1 and adaptor 2) contained long self-complementary stretches that are respectively capable of forming a hairpin-loop structure by self-annealing. Two different sticky ends were introduced into the adaptors. A 80-nt spliceable intronic sequence derived from *BnMS5^a^* [30] of *B. napus* was located in loop2 of adaptor 2, serving as a spacer between the antisense and sense sequences of the resulting inverted repeats, stabilizing the hpRNA DNA clones in bacteria, and triggering RNAi effects in transgenic *B. napus* plants (Step 3; Figure 1). The ligation products among DNA fragments, adaptor 1, and adaptor 2, formed a dumbbell-like structure with a single-stranded closed circle (Step 3; Figure 1). RCA was performed to yield a tandem array of double-stranded inverted repeat DNA units by employing the loop2-specific primer pair loop2-F and loop2-R and Phi29 DNA polymerase (Step 4; Figure 1). RCA products were digested and ligated to the eukaryotic expression vector pBI121, generating the final ihpRNA library expression constructs (Step 5; Figure 1).

### 2.2. RCA-Mediated Construction of PDS ihpRNA

**Figure 2:**
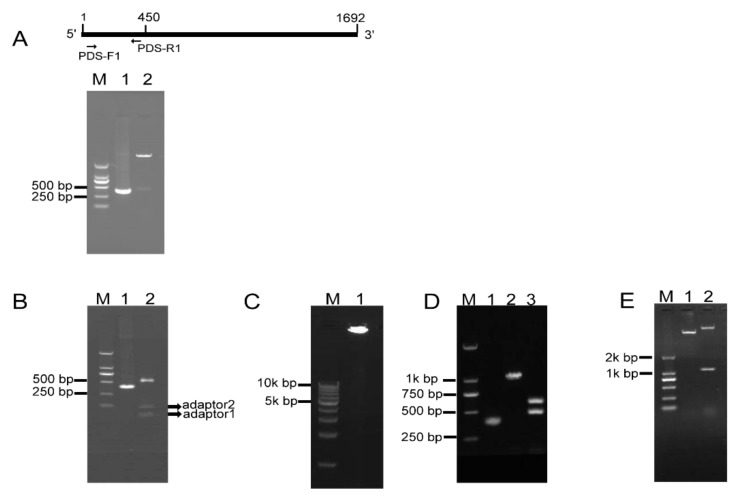
RCA-mediated construction of PDS ihpRNA. (**A**) Amplification and cloning into pKan-*Bsa*I. A 400-bp PDS fragment was amplified using the PDS-F and PDS-R primers (lane 1), followed by T-A cloning. *Bsa*I digestion indicates that a 450-bp PDS fragment was inserted into the pKAN-*Bsa*I vector (lane 2). (**B**) Formation of closed circular molecules. *Bsa*I-digested PDS fragment is shown in lane 1, ligation products between two adaptors and the *Bsa*I-digested PDS fragment are shown in the upper band in lane 2, and free adaptors are shown in the lower bands in lane 2. (**C**) Rolling circular amplification. Closed circular DNA was amplified using Phi29 DNA polymerase and loop2-specific primers loop2-F and loop2-R, resulting in large double DNA molecules containing multiple-unit inverted repeats (lane 1). (**D**) Verification of inverted DNA repeats. The PDS control DNA is shown in lane 1. RCA products were digested with *Bam*HI and *Sac*I, generating a 1.1-kbp fragment in lane 2. *Bam*HI and *Sac*I-digested fragments were further cut using *Bsa*I, generating a 500-bp fragment and a 600-bp fragment (lane 3). (**E**) Construction of the pBI121-*PDS*-ihpRNA. *Bam*HI and *Sac*I-digested PDS fragment of the RCA products was inserted into eukaryotic expression vector pBI121, generating the *PDS*-ihpRNA. The construct was confirmed by *Bam*HI and *Sac*I digestion (lane 1).

To investigate whether RCA effectively generates iphRNA constructs, the *B. napus* phytoene desaturase (*PDS*) gene coding sequence of 450 bp in size (from the initial codon ATG) was amplified using the primer pair PDS-F1/PDS-R1 (Figure 2A), an ‘A’ was added and inserted into the pKan-*Bsa*I using the T-A approach. Following *Bsa*I digestion, PDS fragments with two different sticky ends, respectively, were obtained (Figure 2A), which then were ligated to adaptor 1 and adaptor 2, forming closed circular DNA molecules. Agarose gel electrophoresis of the ligation product showed that almost all of the *Bsa*I-digested PDS DNA fragments were connected to both adaptors, generating adaptor1:PDS DNA::adaptor2 closed circular ligation products (Figure 2B). RCA were performed using the ligation products, Phi29, and the loop2-specific primer pair loop2-F/loop2-R. Large DNA molecules containing inverted repeat units of double-stranded DNA were observed (Figure 2C). The RCA products were confirmed by *Bam*HI and *Sac*I digestion, which generated 1.1-kb of linear fragment that was approximately two-fold greater in size than the unamplified *PDS* control, and by a further digestion for the 1.1-kb fragment with *Bsa*I, which produced a 500-bp fragment, including the *PDS* control and partial sequences of loop1, and a 600-bp fragment, including 450-bp PDS control fragment and 128-bp adaptor 2 (Figure 2D). These results indicated that a pool of single inverted repeat units for *PDS* were formed by RCA. Finally, *Bam*HI- and *Sac*I-digested fragments of RCA products were inserted into eukaryotic expression vector pBI121, generating a 121-PDS-ihpRNA construct (Figure 2E).

### 2.3. Testing of Eukaryotic Expression 121-PDS-ihpRNA Construct

**Figure 3:**
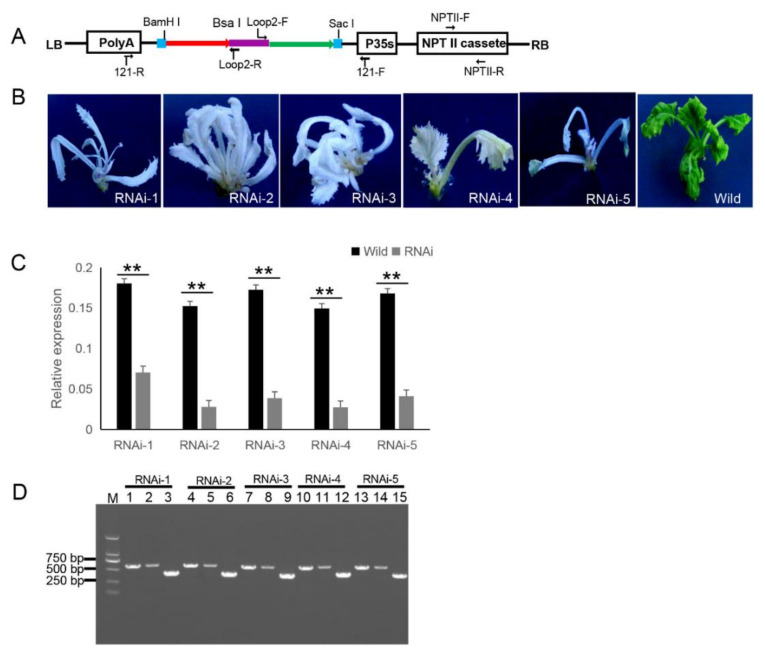
121-PDS-ihpRNA-mediated silencing of the *PDS* gene. (**A**) Map of the binary vector 121-PDS-ihpRNA. NPTII-F and NPTII-R primers were used in the identification of positive transgenic plants, the primer pairs loop2-F/121-F and loop2-R/121-R were utilized to rapidly screen target genes. Blue line is the adaptor1 sequence. Purple line is the adaptor2 sequence. Red line with the arrow is the sense strand of dsDNA. Green line with the arrow is the anti-sense strand of dsDNA. (**B**) Interfering phenotypes of 5 PDS-ihpRNA transgenic plants. RNAi-1, RNAi-2, RNAi-3, RNAi-4, and RNAi-5 were PDS-ihpRNA transgenic plants. Wild was the transgenic negative plant. (**C**) qRT-PCR analysis of the *PDS* target gene mRNA level in the five hpRNA lines shown in (**B**) using the primer pair PDS-F2/PDS-R2. Data shown as mean ± s.d.,**, *p* < 0.05.. (**D**) Integration into the *Brassica napus* genome for the *PDS* ihpRNA expression cassette. For the five PDS RNAi lines, loop2-F and 121-F or loop2-R and 121-R primer pair could amplify the bands with the desired size, demonstrating that the PDS ihpRNA expression cassette was successfully integrated into the *B. napus* plants. Lanes 3, 6, 9, 12, and 15 show the 450-bp PDS control; lanes 1, 4, 7, 10, and 13 exhibit the PCR products of loop2-F and 121-F; lanes 2, 5, 8, 11, and 14 depict the PCR products of loop2-R and 121-R.

The 121-PDS-ihpRNA eukaryotic expressional construct was initially transferred into *Agrobacterium* strain GV3101 by electroporation, which was then transformed into *B. napus* cultivar zhongshuang 6 [31] via *Agrobacterium*-mediated gene transformation [32] to generate transgenic PDS-ihpRNA plants. Currently, this method of genetic transformation is considered to be of high efficiency (about 17%) [33]. The binary vector pBI121 harbors the neomycin phosphotransferase gene (*NPTII*) resistance selection marker (Figure 3A). The selection marker-resistant regenerated T_0_ transformants were initially subjected to kanamycin-based selection and then rooted well in selective medium. The selected resistant T_0_ transformants were then verified by PCR-based screening using the corresponding primer pairs NPTII-F and NPTII-R, which were specific for the *NPTII* gene in the binary construct (Figure 3A). A total of five transgenic plants (approximately 17% transformation efficiency) were obtained, all displaying photo-bleaching phenotypic characteristics (Figure 3B).

Other research has shown that Small RNAs are implicated in post-transcriptional RNA silencing in transgenic plants. RNAs transcribed in RNA silencing transgenic plants form dsRNAs that trigger siRNA biogenesis, thus causing RNA silencing in transgenic plants [34]. In addition, hpRNA by the 35S Pol II promoter can result in the accumulation of predominantly 21-nt siRNAs [35,36,37,38]. To verify whether the *PDS* gene was silenced, qRT-PCR corresponding to the *PDS* target gene in five RNAi T_0_ lines was investigated, which confirmed that the mRNA expression level of the target *PDS* had significantly decreased (Figure 3C). These results suggested that the PDS ihpRNA construct effectively silenced the *PDS* target gene.

Due to the complex secondary hairpin structures formed by ihpRNA constructs, special primers (avoiding specificity to the stem of the hairpin structure) were designed to efficiently amplify the hpRNA target sequence in the binary pBI121 vector. The primer pairs loop2-F and 121-F or loop2-R and 121-R were used to identify the target gene sequences of the ihpRNA lines (Figure 3D). For the five PDS RNAi lines, loop2-F and 121-F or loop2-R and 121-R primer pair successfully amplified bands of the desired size, and the amplification products were sequenced, demonstrating that the *PDS* ihpRNA expression cassette was successfully integrated into the *B. napus* plants (Figure 3D). This result indicated that these two pairs of primers could be employed to rapidly identify the candidate target genes of the ihpRNA lines.

### 2.4. Construction of the Intermediate B. napus cDNA Library

**Figure 4:**
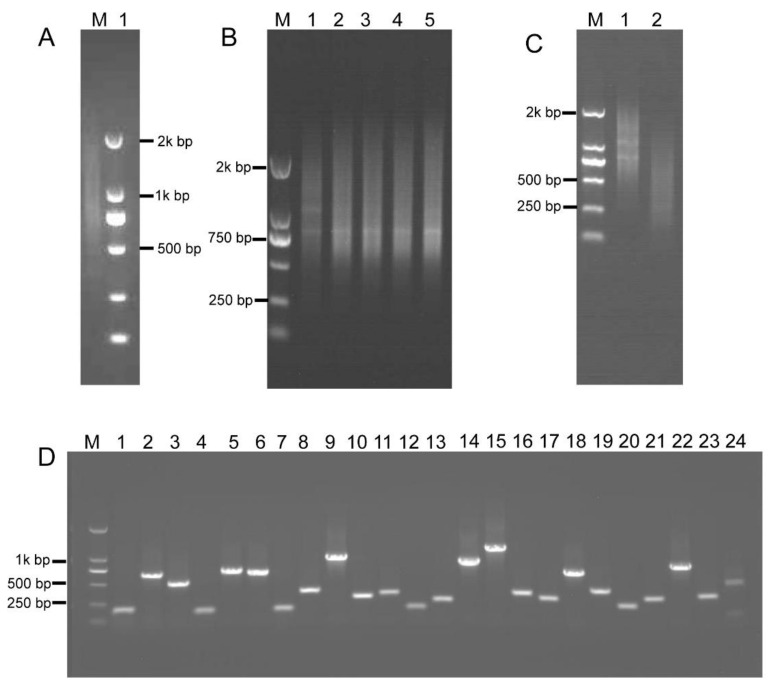
Construction of the intermediate *B. napus* cDNA library. (**A**) First-strand cDNA was synthesized (lane 1). (**B**) Optimization of the conditions for LD-PCR. A range of PCR cycles was performed. Lanes 1–5 correspond to 15, 18, 21, 24, and 27 cycles, respectively. (**C**) dsDNAs were fragmented with restriction enzymes, *Rsa*I, *Bsa*I, *Bam*HI, *Sac*I, *Bgl*II, and *Hin*d III, and fragments 200–800 bp in size were gel-fractionated (lane 2), lane 1 shows dsDNA before digestion. (**D**) Random PCR of inserts in the intermediate cDNA library. Approximately 100% of the clones contained inserts ranging from 200 to 800 bp in size (lanes 1–24).

To investigate most of the *B. napus* transcripts, total RNA was extracted from various tissues at different developmental stages (see Section 4), including roots, stems, leaves, inflorescences, and siliques. The high-quality, first-strand, full-length cDNAs were synthesized using the SMARTer PCR cDNA synthesis kit (Clontech Laboratories Inc., Mountain View, CA, USA) (Figure 4A). Double-stranded cDNA (dscDNA) was obtained by amplification of the first-strand cDNA templates using LD PCR [39]. For LD PCR, overcycled dsDNA resulted in less representative transcripts, whereas undercycling generates lower dsDNA yield. Therefore, a series of cycles, including 15, 18, 21, 24, and 27 cycles, were performed to optimize the number of PCR cycles. Figure 4B shows that the number of LD PCR products reached a plateau at 24 cycles. After 24 cycles, a high-molecular weight smear was detected in the gel, demonstrating that LD PCR was overcycled. Therefore, the optimal number of PCR cycles was 24 (Figure 4B). The dsDNAs were subsequently fragmented using restriction enzymes, and fragments of 200–800 bp in size were gel-fractionated (Figure 4C) and ligated into pKan-*Bsa*I using the T-vector ligation strategy, yielding the intermediate cDNA library that contained 9.2 × 10^6^ clones. About 100% of the clones contained insertional fragments of 200–800 bp in size (Figure 4D). These results indicated that the intermediate library was of high quality, with a relatively low empty-vector rate and was suitable for subsequent ihpRNA library construction.

### 2.5. RCA-Induced ihpRNA Library Construction of B. napus

**Figure 5:**
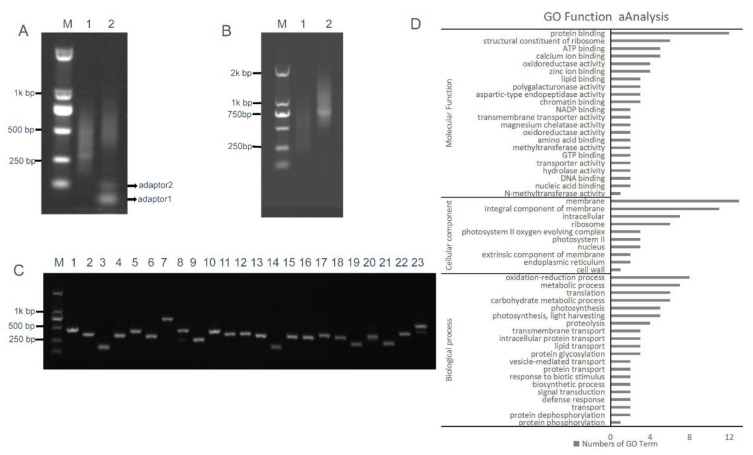
RCA-mediated ihpRNA mutant library construction of *B. napus.* (**A**) The intermediate cDNA library was digested with *Bsa*I, generating DNA fragments of 200–700 bp in size with two different *Bsa*I adhesive termini (lane 1), and purified *Bsa*I-digested cDNA library insertional fragments were ligated with adaptors 1 and 2, leading to closed circular DNA molecules (lane 2). (**B**) The RCA products were digested with *Bam*HI and *Sac*I to release single-unit inverted repeats. DNA fragments of 400–1400 bp, representing the single unit inverted repeats (lane 2), were approximately double that of the unamplified *Bsa*I-digested cDNA control (lane 2) in size. (**C**) Random PCR of inserts in the binary pBI121. Approximately 100% of the clones contained inserts ranging from 200 to 800 bp in size (lanes 1–23). (**D**) Histogram showing Gene Ontology functional analysis of randomly selected insertional fragments. The frequency of GO terms was analyzed using GO Slim Assignment. The y-axis and x-axis indicate the names of clusters and the number of genes in each cluster, respectively.

*Bsa*I-digested intermediate cDNA library insertional fragments with two different *Bsa*I adhesive termini were obtained from plasmid DNA of the intermediate library and were ligated with adaptors 1 and 2, thereby generating closed circular DNA molecules (Figure 5A). RCA was performed using circular DNAs and loop2-specific primers loop2-F and loop2-R. To confirm that RCA yielded linear concatamers of inverted repeat DNAs, the RCA products were analyzed by restriction enzyme digestion. Figure 5B shows that double digestion with *Bam*HI and *Sac*I generated fragments within the expected size range of 400–1600 bp and containing a pool of inverted repeat units, whose size was approximately double that of the unamplified *Bsa*I-digested cDNA control. Fragments digested with *Bam*HI and *Sac*I were then ligated into the binary pBI121 vector. Ligation products were introduced into *Escherichia coli* DH5α cells by electroporation, thereby generating an ihpRNA library consisting of 7.9 × 10^6^ clones. To examine whether insertions in the ihpRNA library contain inverted repeats of diverse target sequences, 23 clones were randomly selected and analyzed by PCR amplification with primer pair loop2-F and 121-F, and almost all of these contained insertional inverted repeats DNAs (Figure 5C).

We further investigated the quality of the ihpRNA library by sequencing a subset of randomly selected clones using loop2-F and loop2-R primers and analyzing sequence data against *B. napus* sequence databases. Of the 200 clones from the ihpRNA library, 192 (96%) had an inverted repeat sequence of >100 bp in length (average size: 300 bp), corresponding to 187 genes or gene families (Table S2). These genes represent a variety of functional categories, including cellular component, biological process, and molecular function (Figure 5D). This finding indicated that the constructed ihpRNA library was of high quality and with a good level of gene coverage.

### 2.6. Generation of the Transgenic ihpRNA Mutant Population of B. napus

**Figure 6:**
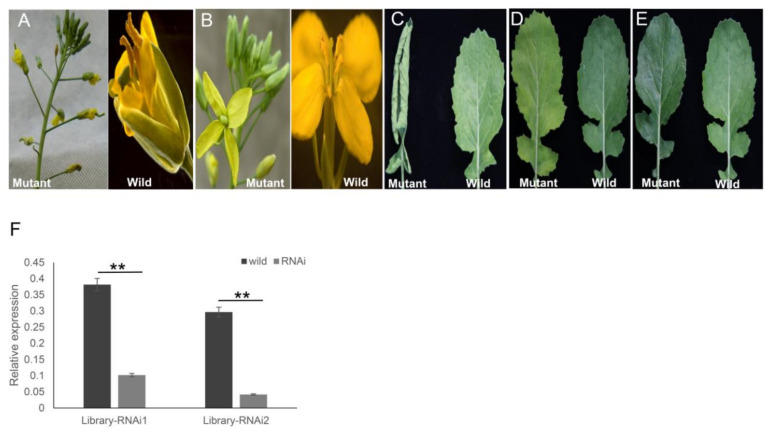
Five representative mutant phenotypes of the ihpRNA transgenic oilseed rape. (**A**) Alterations in the floral model. (**B**) Alterations in the stamen development. (**C**) Curly leaves. (**D**) Yellowed leaves. (**E**) Smooth leaves. (**F**) qRT-PCR analysis of target gene mRNA level in the two ihpRNA lines shown in (A) and (B). Data shown as mean ± s.d., **, *p* < 0.05.

For transformation of *B. napus*, the ihpRNA library was initially introduced into *A. tumefaciens GV3101* by electroporation, thereby generating the corresponding *Agrobacterium* library that contained at least 8.4 × 10^6^ clones. The library was then utilized to transform cultivar Zhongshuang 6 to generate a transgenic ihpRNA population of *B. napus*. Among the 96 positive T_0_ transgenic lines, five ihpRNA lines with observable mutant phenotype, including the abnormal floral model (Figure 6A), male sterility (Figure 6B), smooth leaves (Figure 6C), curly leaves (Figure 6D), and yellowed leaves (Figure 6E), were acquired. The mutants in flower organs, including alterations in the floral model (Figure 6A) and the stamen development (Figure 6B) were named library-RNAi1 and library-RNAi2, respectively. The target gene sequences of library-RNAi1 and library-RNAi2 lines were easily identified by amplification of the ihpRNA sequence using the 121-R/Loop2-R or 121-F/Loop2-F primer pairs and sequenced, showing that the ihpRNA expression cassette was inserted into the genome of *B. napus* (Figure S1). The target gene of library-RNAi1 was one of *BnAP2* family members, which was essential for the determination of the identity of sepals and petals and was a primary member of class A genes, containing three copies in *B.napus* genome (LOC106411443, LOC106445506, and LOC106445506) [32,40]. The hpRNA in library-RNAi1 line matched perfectly with the sequence of LOC106411443 from 11 to 170 nt. The target gene of library-RNAi2 was one of *BnMS5* family members, which was involved in fertility-related traits in *B. napus* [30,41,42]. *BnMS5* gene family members targeted by the hpRNA in library-RNAi2 line included LOC106453110, LOC111208847, LOC106453110, LOC106411501, and LOC106360299. The hpRNA in library-RNAi2 line matched perfectly with the sequence of LOC111208847 from 17–376 nt. As the relative nucleotide sequence identities of the targeted gene members in library-RNAi1 and library-RNAi2 were more than 90%, respectively, primers were designed according to conserved sequences (Table S1). qRT-PCR of the target genes in the two RNAi T_0_ lines were examined, which confirmed that the expression level of the target mRNA significantly decreased, indicating that the hpRNA derived from ihpRNA library construction induced efficient silencing of itself and the related family members (Figure 6F). For the other three mutants, the target sequences needed to be analyzed on the T_1_ or T_2_ generation due to the presence of multiple transgene insertion sites. These findings showed that our ihpRNAi mutant library may potentially be utilized as a powerful tool for gene identification in *B. napus*.

## 3. Discussion

Various ihpRNA constructs employing conventional cloning systems have been extensively employed in functional studies for gene silencing [24]. However, traditional ihpRNA vector construction methods involve specific PCR amplification of target sequences and single or multistep ligation reactions, which were generally tedious and expensive. High-throughput gene silencing, which simultaneously involves the construction of a large number of ihpRNA vectors targeting thousands of genes in a specific plant species, thus remain as a major challenge [28,43]. The RCA strategy provides an effective method of producing an array of tandem template copies, thereby allowing the simultaneous formation of a large number of ihpRNA constructs. Using RCA, long ihpRNA libraries of *Arabidopsis* and rice have been successfully generated [26,27]. In the present study, to improve the efficiency of the RCA system, bidirectional PCR using two loop-specific primers were designed, thereby eliminating noninverted repeats that often result from the use of six-nucleotide random primers [28,43].

The present study selected 200–800 bp of dsDNA fragments for hpRNA construction. We avoided using longer dsDNA fragments because a previous report has shown that inverted DNA repeats with a relatively short spacer and very long stems are unstable in bacteria [44]. In addition, studies have demonstrated that approximately 120 bp fragments could induce efficient gene silence [21,44]. In addition, ihpRNA constructs spliced by introns were more effective in triggering gene silencing in plants [24]. Therefore, a 90 bp intron as the spacer of stems was introduced to generate a *B. napus* ihpRNA library. Furthermore, the intron-specific primer combined with the primer flanking the binary vector could quickly screen candidate target genes or gene families. Using this improved RCA and ihpRNA strategy, we produced a long ihpRNA library with good coverage of *B. napus* genes.

Approximately 3.5% of EMS-mutagenized or T-DNA insertion populations, which generally harbor mutations occur in nonexonic sequences, display visible mutant phenotypes [45], whereas 50% of positive ihpRNA transgenic lines of a rice ihpRNA library targeting exonic sequences exhibit visible phenotypes [27]. For tetraploid *B. napus*, its genome possesses a large number of homologous genes. Simultaneous multigene repression is unlikely to occur through T-DNA or EMS mutagenesis in oilseed rape. The present study generated an ihpRNA construct library from a cDNA library that targeted exonic sequences, thereby providing a cost-effective way of silencing all or most homologous genes or gene family members in *B. napus*. However, previous reports have indicated that detecting conditional or subtle mutant phenotypes is generally difficult [46,47]. The efficiency of detecting invisible mutants largely depends upon how closely or under what conditions these mutants are being investigated. No visible mutant phenotypes were far from confirmation that a gene was nonfunctional or redundant. Several approaches may be utilized to screen for *B. napus* mutant phenotypes, which includes the development of standards of normal development and growth during various growth stages and establishing a set of growth conditions to ease detection of conditional mutant phenotypes.

The ihpRNA library constructs used in this study were driven by a strong constitutive promoter, namely, the CaMV 35S promoter. Alternatively, an inducible or tissue-specific promoter may be utilized in future studies to explore the functions of housekeeping or essential gene functions in future studied genes. In addition, ihpRNA-induced RNAi often knocks down target genes, rather than knocking out gene expression, which restricts its application of gene function requiring only a low level of mRNA accumulation. Moreover, ihpRNAi mutant libraries largely depend on genetic transformation and tissue culture, which can cause somaclonal variations, thereby generating confusing ihpRNA-induced mutant phenotypes. These problems could be resolved by investigating cosegregation between mutant phenotypes and targeting genes in the segregating transgenic population. As RNAi is a homology dependent process, another major drawback of gene silencing is that it suppresses unintended genes which are similar in sequence to the targeted silencing genes, but have an ectopic phenotype. This issue can be figured out by selection of a unique region of the target sequence.

## 4. Materials and Methods

### 4.1. Construction of the pKAN-BsaI Vector and Cloning of the PDS Gene Fragment

Two oligonucleotides with 5’ phosphorylation were synthesized, including the sense strand pKan-*Bsa* (+) and the antisense strand pKan-*Bsa* (-), as shown in Table S1. Two *Bsa*I sites flanking *Xcm*I sites were introduced into the two oligonucleotides. The two oligonucleotides were used in the amplification of the pKAN vector, and the amplification products were self-ligated to form the intermediate vector pKan-*Bsa*I. The improved intermediate cloning vector Pkan-*Bsa*I contained two *Xcm*I sites, two *Bsa*I sites flanking each *Xcm*I site, and the ccdB lethal gene. *Xcm*I digestion generated two 3’-T overhangs, transforming pKan-*Bsa*I into a T-ended vector that would facilitate the insertion of double strand cDNAs (dscDNAs). The lethal ccdB gene could avoid the recovery of *E. coli* clones with empty vectors. The recognition sequence of *Bsa*I is GGTCTC(N)_1–5_, and the two *Bsa*I sites within the pKan-*Bsa*I vector were ‘GGTCTCGACCT’ and ‘GGTCTCTGGGA’.

The 450-bp coding sequence of the *PDS* gene was isolated from cDNA of *B. napus* cultivar Zhongshuang 6 (Oil Crops Research Institute, CAAS, Wuhan, China) using high fidelity Pfu, to which an ‘A’ was attached. The PDS fragment was separated in a 1.5% agarose gel, excised, and purified, and then inserted into the pKan-*Bsa*I vector using the T-A cloning strategy. The ligation products were then transformed into *Escherichia coli* DB3.1, and the resultant colonies were randomly selected and sequenced.

### 4.2. B. napus Intermediate cDNA Library Construction

*B. napus* Zhongshuang 6 plants were planted under normal farming conditions in the field of the Oil Crops Research Institute of the Chinese Academy of Agricultural Sciences in Hubei, China. Various tissues at different developmental stages were collected, namely, roots, stems, and leaves at the seedling stage; inflorescences at the flowering stage; and siliques at 10, 15, 20, 25 and 30 days after pollination. Total RNA was extracted from these tissues, respectively, using TRIzol^®^ (Invitrogen, Carlsbad, CA, USA) and mixed at equal amounts. cDNA was synthesized using the SMARTer PCR cDNA synthesis Kit according to the SMART cDNA library manual (Clontech Laboratories Inc., Mountain View, CA, USA). dsDNA products of long distance PCR were digested using *Rsa*I, *Bgl*II, *Hind*III, *BamH*I, *Sac*I and *Bsa*I, resulting in fragments 200–800 bp in length, and then treated using T4 DNA polymerase to generating blunt termini. ‘A’ tails were attached to the 3’ termini using Taq DNA polymerase. The DNA fragments were then purified and ligated to a pKan-*Bsa*I vector using the T-A strategy, generating an intermediate cDNA library that consisted of a total of 9.2 × 10^6^ clones.

### 4.3. The Rolling Circle Amplification (RCA)

Two oligonucleotides with 5’ phosphorylation, namely, adaptor 1 and adaptor 2, were synthesized, as shown in Table S1. The oligos were annealed as previously described [48], forming the stem-loop structure. Adaptor 1 carried a 5’ overhang ‘AGGT’ that was complementary to the ‘ACCT’ terminus of *Bsa*I-digested DNA insertion fragments, whereas adaptor 2 harbored a 5’ overhang ‘GGGA’ end that was complementary to the ‘TCCC’ terminus of the digested DNA fragments. *BamH*I and *Sac*I sites were introduced into the loop of adaptor 1, which was convenient for subsequent connection of the inverted repeat units to the eukaryotic expression vector.

For generation of closed circular DNA molecules, the insertional fragments were excised from the pKan-*Bsa*I vector by *Bsa*I digestion followed by gel purification, and about 400 ng of the purified DNA fragments was mixed with 200 ng each of adaptor 1 and adaptor 2, 3 µL of T4 DNA ligase (NEB, England), and 3 µL of ligation buffer in a total reaction volume of 30 µL. Following overnight incubation at 16 °C, the ligation products, namely, closed circular DNAs, were purified and dissolved in 100 µL of sterile water.

For RCA, 30 µL of the ligation reaction products was mixed with 10 µL of Phi29 buffer and 5 µL each of 10 µM loop2-specific primers, namely, loop2-F and loop2-R (Table S1). The mixture was heated at 95 °C for 5 min and chilled on ice for 15 min, to which 5 µL of 10 mM dNTPs, and 2 µL of Phi29 polymerase (Fermentas, Lithuania) were added. After 6–8 h of incubation at 30 °C, the amplified DNAs were precipitated with 0.1 volume of 3 M sodium acetate and 2.5 volumes of ethanol, and dissolved in 40 µL of sterile water.

### 4.4. ihpRNA Library Construction

To generate closed circular cDNAs, the intermediate cDNA library was fragmented with *Bsa*I, and the insertion dsDNA fragments were gel-purified and ligated to adaptor 1 and adaptor 2. The closed circular cDNAs were amplified by RCA using loop2-specific primers (loop2-F and loop2-R), yielding linear concatamers of inverted repeat DNAs. The RCA products were digested using *BamH*I and *Sac*I and then ligated into the binary vector pBI121. The ligation products were transformed into *Agrobacterium tumefaciens* strain GV3101 by electroporation, generating an ihpRNA library consisting of 7.9 × 10^6^ clones.

### 4.5. B. napus Transformation

*Agrobacterium*-induced transformation of *B. napus* was conducted as described elsewhere [49]. Kanamycin-resistant plantlets were planted in a plant growth room. The growth conditions were 20 ± 2 °C under a 16 h/8 h photoperiod at a light intensity of 44 μmol m^−2^·s^−1^ and 60–90% relative humidity. Kanamycin-positive transformants were further verified by PCR using primers specific to the *Npt*II gene (Npt-F, Npt-R). The primers used in amplification are listed in Table S1.

### 4.6. Quantitative Real-Time PCR (qRT-PCR)

The extracted total RNA was initially treated with *DNa*seI to remove any genomic DNA contamination and then used as template for cDNA synthesis with a reverse transcription kit (Promega Corp., WI, USA) according to the manufacturer’s recommendations. qRT-PCR was performed using an Applied Biosystems Prism 7500 Analyzer and GoTaq qPCR Master Mix (Promega Corp., WI, USA). Three independent biological and technical replicates were used for each plant. The results were analyzed using the 2^−^^ΔΔ^^CT^ method [50]. The actin gene of *B. napus* was used as internal control for normalization. The primers used are listed in Table S1.

### 4.7. Gene Ontology (GO) Function Analysis of Randomly Selected Genes

GO is a usual standardized gene analysis classification system which categorizes genes and gene products in terms of their biological process, cellular component, and molecular function. Gene Ontology (GO) is an international standardized gene functional classification system that describes properties of genes and their products in any organism, containing three ontologies: molecular function, cellular component, and biological process. A total of 99 genes from the ihpRNA library belonging to 52 categories were found (Table S3). These genes were involved in biochemistry, metabolism, development, and so on.

## Supplementary Materials

Supplementary materials can be found at https://www.mdpi.com/1422-0067/21/19/7243/s1. Figure S1: The ihpRNA sequences of library-RNAi1 and library-RNAi2. Blue bases are vector sequences used for rapid identificaion of target genes. Red and green bases are inverted repeats of target genes. Purple bases are the intron sequence. Table S1: DNA oligonucleotide sequences; Table S2: 187 genes of randomly selected clones from the ihpRNA library; Table S3: GO terms of 99 genes from the ihpRNA library.

## Abbreviations

ihpRNA	Long intron-spliced hairpin RNA
RCA	Rolling circle amplifica
RNAi	RNA interference
PTGS	Post-transcriptional gene silencing
dsRNA	Double-stranded RNA
siRNA	Small interfering RNA
RISCs	RNA-induced silencing complexes
PDS	Phytoene desaturase
